# Nanohybrid Composites Based on TiO_2_ and Single-Walled Carbon Nanohorns as Promising Catalysts for Photodegradation of Amoxicillin

**DOI:** 10.3390/molecules28196958

**Published:** 2023-10-06

**Authors:** Radu Cercel, Andreea Androne, Cristina Stefania Florica, Adam Lőrinczi, Constantin Serbschi, Mihaela Baibarac

**Affiliations:** 1 National Institute of Materials Physics, Atomistilor Street 405A, 077125 Bucharest, Romania; radu.cercel@infim.ro (R.C.); andreea.radu@infim.ro (A.A.); stefania.florica@infim.ro (C.S.F.);; 2 Faculty of Physics, University of Bucharest, Atomistilor Street 405, 077125 Magurele, Romania; 3 Bioelectronic SRL, Cercelus Street 54, 100028 Ploiesti, Romania; bioelectronic90@yahoo.com

**Keywords:** titanium dioxide, reduced graphene oxide, acetaminophen, photocatalytic properties

## Abstract

In this work, applications of nanohybrid composites based on titanium dioxide (TiO_2_) with anatase crystallin phase and single-walled carbon nanohorns (SWCNHs) as promising catalysts for the photodegradation of amoxicillin (AMOX) are reported. In this order, TiO_2_/SWCNH composites were prepared by the solid-state interaction of the two chemical compounds. The increase in the SWCNH concentration in the TiO_2_/SWCNH composite mass, from 1 wt.% to 5 wt.% and 10 wt.% induces (i) a change in the relative intensity ratio of the Raman lines located at 145 and 1595 cm^−1^, which are attributed to the E_g_(1) vibrational mode of TiO_2_ and the graphitic structure of SWCNHs; and (ii) a gradual increase in the IR band absorbance at 1735 cm^−1^ because of the formation of new carboxylic groups on the SWCNHs’ surface. The best photocatalytic properties were obtained for the TiO_2_/SWCNH composite with a SWCNH concentration of 5 wt.%, when approx. 92.4% of AMOX removal was achieved after 90 min of UV irradiation. The TiO_2_/SWCNH composite is a more efficient catalyst in AMOX photodegradation than TiO_2_ as a consequence of the SWCNHs’ presence, which acts as a capture agent for the photogenerated electrons of TiO_2_ hindering the electron–hole recombination. The high stability of the TiO_2_/SWCNH composite with a SWCNH concentration of 5 wt.% is proved by the reusing of the catalyst in six photodegradation cycles of the 98.5 μM AMOX solution, when the efficiency decreases from 92.4% up to 78%.

## 1. Introduction

Amoxicillin (AMOX) is an organic compound used as an antibiotic in the treatment of various infections, such as urinary tract [1], dental [2], chest [3], sinus [4], etc. This organic compound is found in drugs commercialized under different names, including (i) Amoksiklav (AMOX-drug), Augmentin, Amoclan, when AMOX is mixed with clavulanate potassium; (ii) Voquezna Triple Pak, which contains a mixture of AMOX, clarithromycin and vonoprazan; (iii) Omeclamox-Pak, when active compounds consist of AMOX, clarithromycin and omeprazole; and (iv) Prevpac, when the ingredients include AMOX, clarithromycin and lansoprazole. One of the main pollutants reported in medical wastewater, groundwater or surface water are antibiotics. AMOX is not effectively removed by traditional organic wastewater treatment methods (for example electrolysis, activated carbon adsorption, biological methods, etc.) [5], which is why advanced oxidation processes were widely used when its complete oxidation was reported [6]. Thus, the methods of ozonation, Fenton, photo-Fenton and photocatalysis in the presence of semiconductors proved to be alternatives to the conventional methods for the degradation of AMOX into biodegradable compounds, CO_2_ and H_2_O [7,8,9,10,11]. At present, the AMOX concentration reported in surface water and secondary water drainage was at the μg L^−1^ order [12]. Among the most used catalysts for the AMOX photodegradation are (i) TiO_2_ [13]; (ii) TiO_2_ nanotubes/graphene [14]; (iii) ZnO/TiO_2_ nanocomposites [15]; (iv) TiO_2_/reduced graphene oxide [16], nanodiamond/TiO_2_ composites [17], ZnO nanoparticles with hexagonal and spherical morphology [18]; etc. The best efficiency of AMOX photodegradation, equal to 99.84%, was reported when the TiO_2_/graphene oxide composites were used as catalyst [19]. A lower AMOX photodegradation efficiency of only 95% has been reported for catalysts such as TiO_2_/graphene composites after a 3 h exposure time to UVA light [14]. 

In contrast with this progress, a new catalyst for the AMOX photodegradation based on TiO_2_ and single-wall carbon nanohorns (SWCNHs) is reported. Catalyst efficiency based on TiO_2_/SWCNH composites for the photodegradation of a 98.5 μM AMOX solution equal to 92.4% will be reported to be achieved after exposure for 90 min to UVC light. At present, the main synthesis methods of the TiO_2_/SWCNH composites are (a) the metal–organic chemical vapor deposition, when the nano-petals based on TiO_2_ and SWCNH results [20]; (b) the hydrolysis of tetrabutoxytitanium in the presence of SWCNHs [21]; and (c) a solvothermal process [22]. In this work, we will use the solid-state interaction of TiO_2_ and SWCNHs in order to obtain TiO_2_/SWCNH composites. The characterization methods used in the case of the TiO_2_/SWCNH composites were X-ray diffraction, Raman scattering, scanning electron microscopy, X-ray photoelectron microscopy, UV-VIS spectroscopy and photoluminescence (PL). In this work, the optical and structural properties of the TiO_2_/SWCNH composites will be highlighted by Raman scattering, FTIR spectroscopy and X-ray diffraction.

The following applications were reported for the TiO_2_/SWCNH composites: (a) hydrogen production [22], (b) lithium–ion batteries [21] and (c) the photodegradation of phenols [20]. In the present work, we will demonstrate that TiO_2_/SWCNH composites can also be used as a catalyst for AMOX photodegradation. Special attention will be given to (i) the concentration of the composite aqueous solution, (ii) the concentration of SWCNHs in the mass of the composite, (iii) the concentration of the AMOX aqueous solution, (iv) the presence of quenchers in the colloidal dispersion, and finally, (v) the reaction kinetics of AMOX aqueous solutions containing the TiO_2_/SWCNH composite. There is currently no information about the stability of this catalyst through its reuse during the photodegradation of pollutants. Therefore, in the present work, the TiO_2_/SWCNH composites’ stability after their reuse in several photodegradation cycles of AMOX will be shown, too. The removal of the TiO_2_/SWCNH composites after AMOX photodegradation will be carried out using PVC membranes prepared according to Ref. [23]. The performance of the TiO_2_/SWCNH catalysts on the photodegradation of commercial pharmaceutical compounds containing AMOX, e.g., Amoksiklav, will also be proved. 

## 2. Results and Discussion

### 2.1. Optical and Structural Properties of the TiO_2_/SWCNH Composites

**Figure 1:**
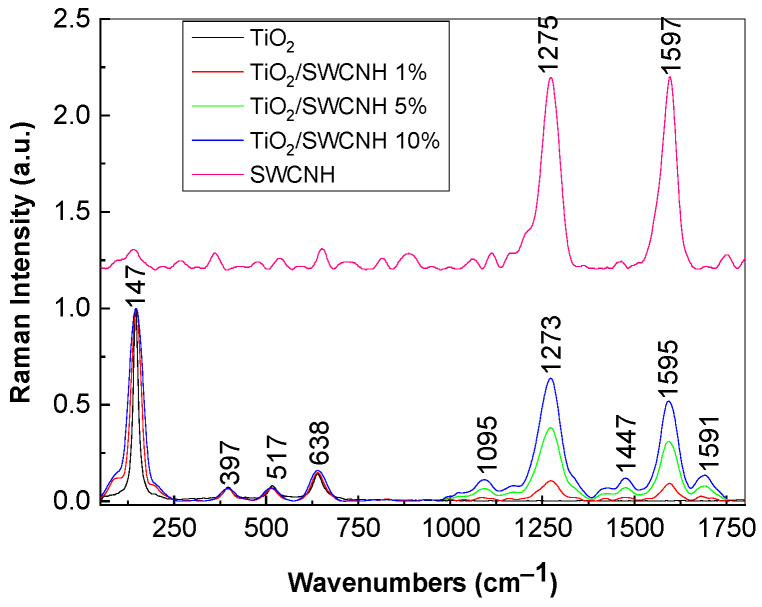
Raman spectra of TiO_2_ with anatase crystalline-phase (black curve), SWCNHs (magenta curve) and the TiO_2_/SWCNH composites, with a SWCNH concentration equal to 1 wt.% (red curve), 5 wt.% (green curve) and 10 wt.% (blue curve).

Figure 1 shows the Raman spectra of TiO_2_, SWCNHs and the TiO_2_/SWCNH composites with a SWCNH concentration equal to 1 wt.%, 5 wt.% and 10 wt.%. TiO_2_ with anatase crystalline phase highlights Raman lines peaked at ~147, 397, 517 and 638 cm^−1^, which were assigned to the following vibrational modes: E_1g_, B_1g_, A_1g_ and E_3g_, respectively [24]. SWCNHs show two Raman lines situated at 1275 and 1597 cm^−1^, often labelled as D and G bands, which were attributed to SWCNH defects and graphitic structures, respectively. The analysis of the Raman spectrum of SWCNHs (magenta curve in Figure 1) indicates that the ratio between the intensities of the D and G bands (I_D_/I_G_) is equal to 1 [25]. According to our expectations, as the SWCNH concentration in the TiO_2_/SWCNH composites mass increases, a growth in the ratio between the D and G bands intensity occurs, as shown by the red, green and blue curve in Figure 1. In the presence of TiO_2_, the value of the I_D_/I_G_ ratio becomes equal to 1.1, 1.23 and 1.3 (red, green and blue curves in Figure 1). This fact indicates an increase of defects weight in the SWCNHs’ structure as a consequence of the interaction between the two constituents. An increase in the full width at half maximum (FWHM) of the Raman line of TiO_2_ peaking at 147 cm^−1^ when a SWCNH concentration in the TiO_2_/SWCNH composites mass increases from 1 wt.% to 10 wt.% is observed. Raman lines peaking at 1095, 1477 and 1691 cm^−1^ were assigned to the vibrational modes of the -C-O-C- and C-C bonds in aromatic ring and C=O stretching [26]. The presence of Raman lines at 1095, 1477 and 1691 cm^−1^ indicates an interaction of SWCNHs with TiO_2_, which leads to the appearance of C-O-C and C=O groups on the surface of SWCNHs.

**Figure 2:**
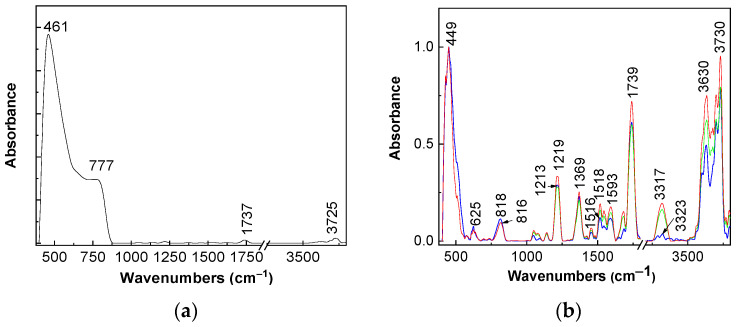
FTIR spectra of TiO_2_ (**a**) and the TiO_2_/SWCNH composites with a SWCNH concentration equal to 1 wt.% ((**b**), blue curve), 5 wt.% ((**b**), green curve) and 10 wt.% ((**b**), red curve).

Complementary information about the SWCNH-TiO_2_ interaction is shown by FTIR spectroscopy in Figure 2. The FTIR spectrum of TiO_2_ with anatase crystalline phase (Figure 2a) is characterized by a band of high absorbance at 457 cm^−1^ that is accompanied by another one at 779 cm^−1^ and other two bands of low intensities at 1737 and 3725 cm^−1^ that are assigned to the stretching vibrational modes of the Ti-O and Ti-O-Ti bonds, H_2_O molecules and the OH bonds coming from the water adsorbed onto the TiO_2_ surface [16,27]. Figure 2b shows the FTIR spectra of TiO_2_/SWCNH composites with a SWCNH concentration equal to 1 wt.%, 5 wt.% and 10 wt.%. Regardless of SWCNH concentration, the following changes are observed in Figure 2a,b: (i) the disappearance of the IR band at 779 cm^−1^; (ii) a down-shift of the IR band from 461 cm^−1^ to 449 cm^−1^ and (iii) an up-shift of the IR bands from 3725 cm^−1^ and 1737 cm^−1^ to 3730 cm^−1^ and 1739 cm^−1^ simultaneous with their absorbance growth. In addition, Figure 2b highlights new IR bands at 816–818, 1213–1219, 1369, 1516–1518, 1593 and 3317–3323 cm^−1^, which were assigned to the following vibrational modes: first order A_2_ [28], C-OH deformation [29], the deformation of the C-C bond of the pentagonal ring [29], E_1u_ of the graphitic lattice [28], and the deformation of the O-H bond [29]. IR bands peaking at 625 and 3630 cm^−1^ were assigned to the vibrational modes of the deformation of aromatic ring of SWCNHs and free OH groups [26]. A puzzling fact is that an IR band peaked at 1730 cm^−1^, which was no longer situated of the IR band at 1739 cm^−1^, as shown in Figure 2b. This IR band was assigned to the C=O stretching vibrational mode of carboxylic groups present on the surface of SWCNHs [30]. The presence of the C=O and C-OH groups on the surface of SWCNHs can be explained only if we accept that the interaction of SWCNHs with TiO_2_ involves a charge transfer, which leads to the appearance of carboxylic groups on the surface of SWCNHs.

**Figure 3:**
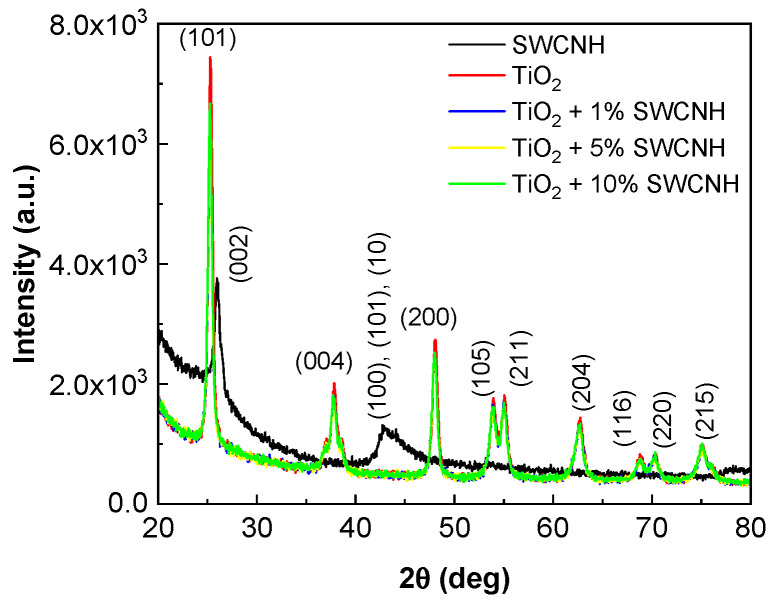
XRD diagrams of SWCNH (black curve), TiO_2_ (red curve) and the TiO_2_/SWCNH composites with a SWCNH concentration equal to 1 wt.% (blue curve), 5 wt.% (yellow curve) and 10 wt.% (green curve).

Figure 3 shows the X-ray diffraction (XRD) diagrams of TiO_2_, SWCNHs and the TiO_2_/SWCNH composites with a SWCNH concentration equal to 1 wt.%, 5 wt.% and 10 wt.%. 

The main XRD peaks of TiO_2_ are situated at 25.3°, 37.8°, 48°, 53.9°, 55.1°, 62.6°, 68.8°, 70.5° and 75.1°. They are assigned to the following crystalline planes of the anatase phase: (101), (004), (200), (105), (211), (204), (116), (220) and (215). This is in accordance with JCPDF no. 00-021-1272. The XRD peaks of SWCNHs are observed at 26° and 43°, the first one being attributed to the crystalline plane (002), while the second one is a combination of the reflections of the crystalline plane (100), (101) and the tubular/turbostatic state of graphitic lattice [31]. One may observe that the TiO_2_/SWCNH composites are keeping the general features of the TiO_2_ polycrystalline powder in regard to the positions of the diffraction peaks. The slight decrease in intensity for the composites compared to the pure TiO_2_ can be understood as a plain addition effect, where SWCNHs fill in the spaces between TiO_2_ crystallites, thus absorbing some of the X-rays. Due to this geometrical arrangement, the structural signature of the SWCNHs will be practically lost in comparison with pure SWCNH powder. In consequence, it is reasonable to state that the addition of SWCNHs will not modify the crystalline structure of TiO_2_; rather, they simply wrap around the TiO_2_ crystallites and fill the space between them.

### 2.2. Photocatalytic Properties of the TiO_2_/SWCNH Composites

When using TiO_2_/SWCNH composites as catalysts for AMOX photodegradation, their efficiency can be calculated with the following equation:

$$D_{\mathrm{eff}}=\left(\frac{A_{0}-A_{t}}{A_{0}}\right)\times 100$$

where *A_0_* and *A_t_* are the absorbances of the band localized at 258–276 nm when the samples were exposed for 0 min and 90 min, respectively, to UV light. To reach the equilibrium of AMOX adsorption/desorption on the catalyst’s surface, before the exposure of samples to UV light, the UV-VIS spectra were recorded for 30 min in the absence of light.

**Figure 4:**
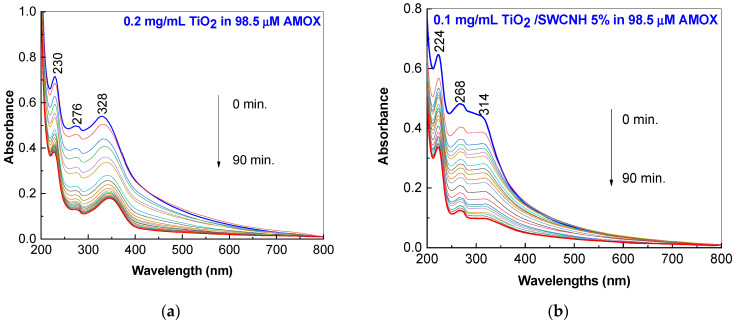

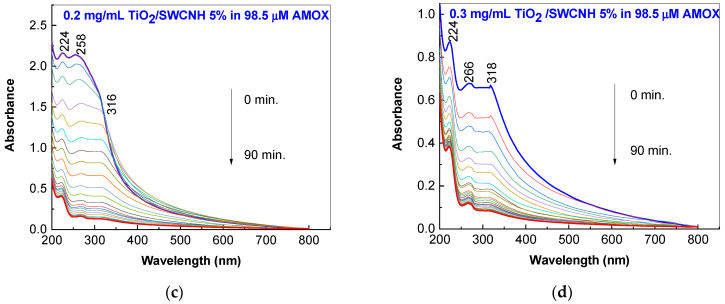
UV-VIS spectra of 98.5 μM AMOX aqueous solution containing 0.2 mg/mL TiO_2_ (**a**) and the TiO_2_/SWCNH composites with a SWCNH concentration of 5 wt.%, recorded during UV irradiation for 90 min. The concentration of the TiO_2_/SWCNH composites in the 98.5 μM AMOX solution were 0.1 mg/mL (**b**), 0.2 mg/mL (**c**) and 0.3 mg/mL (**d**).

Figure 4a,c shows the UV-VIS spectra of the 98.5 μM AMOX solution containing 0.2 mg/mL of TiO_2_ or the TiO_2_/SWCNH composite with a SWCNH concentration equal to 5 wt.%. Figure 4a highlights three bands with maxima at 230 nm, 276 nm and 328 nm, which were assigned to π–π* electronic transition in the AMOX aromatic ring [31], the amide n–π* electronic transition in the AMOX β–lactam chromophore group [31], and TiO_2_ [32]. The exposure of AMOX and TiO_2_ samples to UV light induces a progressive decrease in absorbance as the irradiation time gradually increases up to 90 min. The efficiency of TiO_2_ (0.2 mg/mL) on the photodegradation of the 98.5 μM AMOX aqueous solution is 74.2%. This is a similar behavior that can be observed in the presence of 0.2 mg/mL TiO_2_/SWCNH composite with an SWCNH concentration of 5 wt.% in which the efficiency of this catalyst on the photodegradation of the 98.5 μM AMOX aqueous solution is of 92.4%. According to Figure 4b,d, the efficiency of AMOX photodegradation in the presence of 0.1 mg/mL and 0.3 mg/mL TiO_2_/SWCNH composites in the 98.5 μM AMOX aqueous solution is equal to 75.1% and 82.6%.

**Figure 5:**
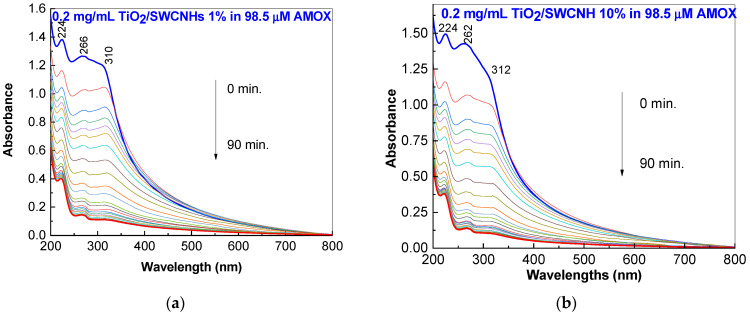
UV-VIS spectra of 98.5 μM AMOX solution containing 0.2 mg/mL TiO_2_/SWCNH composites with a SWCNH concentration of 1 wt.% (**a**) and 10 wt.% (**b**) during UV irradiation for 90 min.

The influence of SWCNH concentration in the TiO_2_/SWCNH composites mass on AMOX photodegradation is analyzed by comparing the results shown in Figure 3b with those presented in Figure 5. As the SWCNH concentration in the TiO_2_/SWCNH composite mass increases from 1 wt.% to 5 wt.% and 10 wt.%, the photodegradation efficiency of 98.5 μM AMOX aqueous solution varies from 88.5% to 92.4% and 90.3%, respectively.

**Figure 6:**
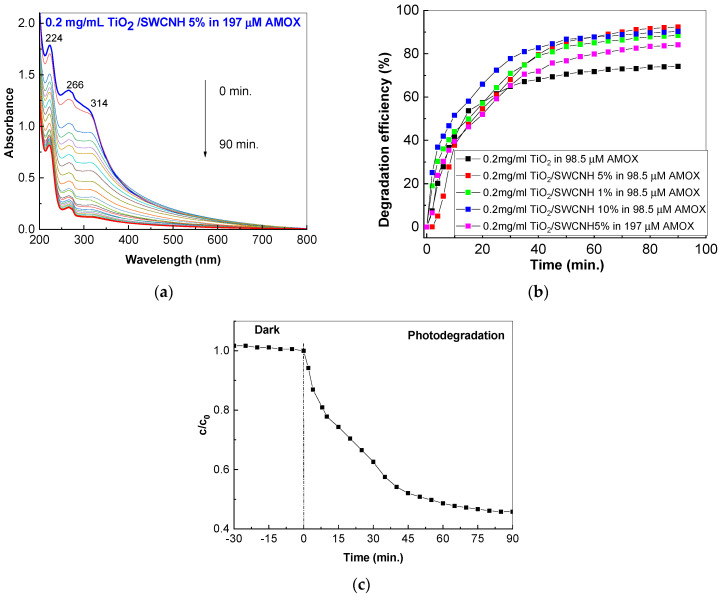
UV-VIS spectra of 197 μM AMOX aqueous solutions containing 0.2 mg/mL TiO_2_/SWCNH composites with a SWCNH concentration of 5 wt.% (**a**). Figure (**b**) shows the degradation efficiency variation of 98.5 μM AMOX solution in the presence of 0.2 mg/mL TiO_2_ (black symbols) and 0.1 mg/mL (red symbols), 0.2 mg/mL (blue symbols) and 0.3 mg/mL (green symbols) of TiO_2_/SWCNH composites with a SWCNH concentration of 5 wt.% after subsequent UV irradiation. The magenta symbols show the photodegradation efficiency of 197 μM AMOX solution containing 0.2 mg/mL of TiO_2_/SWCNH composite with a SWCNH concentration of 5 wt.% after subsequent UV irradiation. Figure (**c**) shows the variation of the c/c_0_ ratio during the photocatalytic degradation of 197 μM AMOX aqueous solutions containing 0.2 mg/mL TiO_2_/SWCNH composites with a SWCNH concentration of 5 wt.% in dark condition and under UV light.

According to Figure 6, the efficiency of the TiO_2_/SWCNH composite (with a SWCNH concentration of 5 wt.%) on the photodegradation of the 197 μM AMOX aqueous solution is 84.1%. According to Figure 6c, the c/c_0_ ratio does not show variations in the dark environment, while under UV light, a significant decrease is reported.

**Figure 7:**
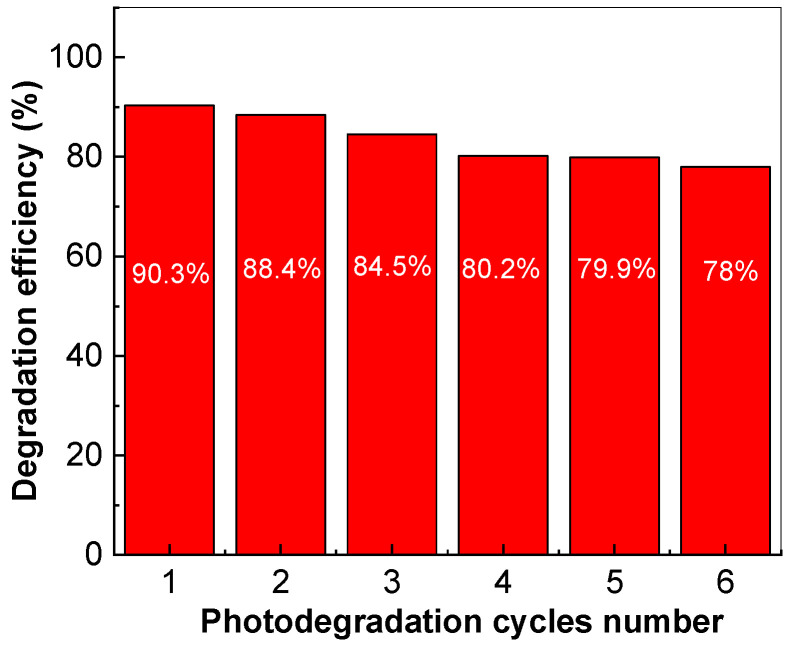
The variation of the efficiency of 0.2 mg/mL TiO_2_/SWCNH composite, with a SWCNH concentration equal to 10 wt.%, on the photodegradation of 98.5 μM AMOX aqueous solution after using the catalyst in six photodegradation cycles.

Information regarding the reuse of the TiO_2_/SWCNH composite (with a SWCNH concentration of 10 wt.%) after six photodegradation cycles of the 98.5 μM AMOX solution is presented in Figure 7.

After six cycles of the photodegradation of 98.5 μM AMOX containing the TiO_2_/SWCNH composite catalyst with a SWCNH concentration equal to 10 wt.%, the photodegradation efficiency is 78%. A 12.3% decrease in the efficiency of AMOX photodegradation occurs when the TiO_2_/SWCNH composite, with an SWCNH concentration equal to 10%, is used as catalyst, which highlights the good stability of this catalyst and its ability to be reused in the photodegradation processes of waters polluted with such pharmaceutical compounds. An explanation for this behavior must take into consideration that the TiO_2_/SWCNH composite can absorb the full spectrum of incident light, like TiO_2_ [33], thereby generating heat at the interface of the TiO_2_/SWCNH composite because the SWCNH powder is black. One consequence of this heat generation is that it will increase the migration of the electron–hole pair into the mass of the TiO_2_/SWCNH composite, thus inducing an increase in charge separation that leads to a greater photocatalytic effect than that of TiO_2_ [34].

**Figure 8:**
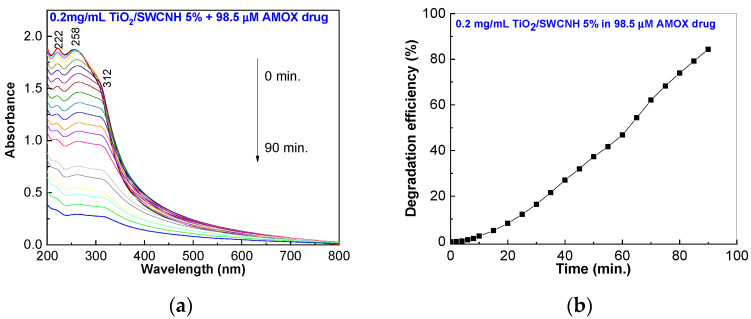
UV-VIS spectra (**a**) and photodegradation efficiency (**b**) of the aqueous solution of Amoksiklav containing 98.5 μM AMOX and 25 μM CLAK and 0.2 mg/mL TiO_2_/SWCNH composite with a SWCNH concentration of 5 wt.% during UV irradiation for 90 min.

The performance of the TiO_2_/SWCNH composite on the photodegradation of the solution containing the pharmaceutical compound Amoksiklav is presented in Figure 8. For this purpose, a solution of AMOX and CLAK (sample labeled as AMOX-drug) was prepared using the drug commercialized as Amoksiklav.

**Figure 9:**
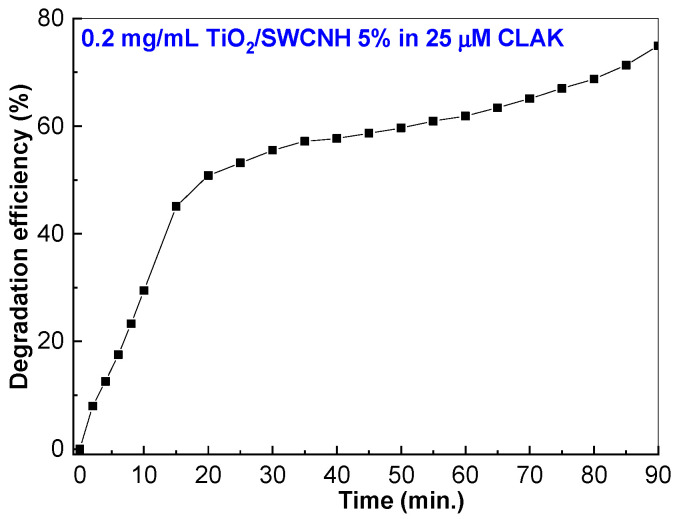
The variation of the photodegradation efficiency of the 25 μM CLAK aqueous solution containing 0.2 mg/mL of the TiO_2_/SWCNH composite, with a SWCNH concentration of 5 wt.%, during UV irradiation for 90 min.

The shape of the UV-VIS spectra shown in Figure 8a, as well as their behavior under UV irradiation, are similar to those of Figure 4c. According to Figure 8b, the efficiency of the TiO_2_/SWCNH composite, with a SWCNH concentration equal to 5 wt.%, on the photodegradation of Amoksiklav solution containing 98.5 μM AMOX and 25 μM CLAK is 84.3%, a value which is lower than that of the sample prepared from AMOX coming from the Sigma-Aldrich company (92.4%) (Burlington, MA, USA). An explanation for this result must consider the CLAK photodegradation in the presence of the TiO_2_/SWCNH composite with a SWCNH concentration equal to 5 wt.%. To support this statement, Figure 9 shows the variation of photodegradation efficiency of 25 μM CLAK solution, prepared from the powder from the Sigma-Aldrich company, in the presence of TiO_2_/SWCNH composite (with a SWCNH concentration of 5wt.%) under UV irradiation for 90 min.

According to Figure 9, after a 90 min UV irradiation period of the CLAK aqueous solution containing the TiO_2_/SWCNH composite, the efficiency of the CLAK photodegradation is 75%.

The photodegradation mechanism of AMOX in the presence of the TiO_2_ catalyst was published in 2010 [35]. This mechanism involves the generation of hydroxyl radicals (•OH) and superoxide ion radicals (•O_2_^−^), which can interact with AMOX, resulting in degradation products. A similar mechanism must also be considered for the TiO_2_/SWCNH composite. Thus, under UV light, holes and electrons are generated in the valence band and conduction band, respectively, of TiO_2_. The transfer of electrons from the conduction band of TiO_2_ to SWCNHs will allow for successive interactions with O_2_ existing in the AMOX aqueous solution, leading to the formation of reactive oxygen species. The generation of hydroxyl radicals occurs when the holes in the valence band of TiO_2_ interact with H_2_O. Subsequently, hydroxyl radicals, being unstable species, will interact with AMOX, leading to the photodegradation products reported in Ref. [35]. 

**Figure 10:**
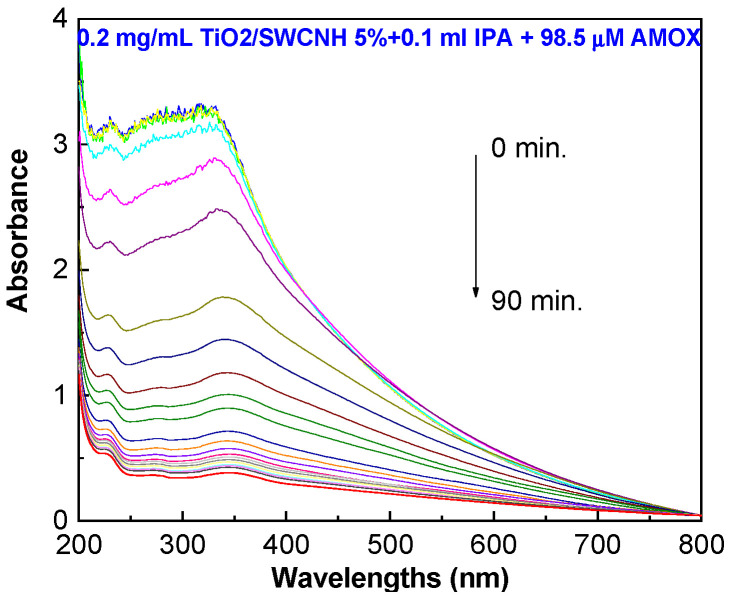
UV-VIS spectra of 98.5 μM AMOX solution containing 0.2 mg/mL of the TiO_2_/SWCNH composite (with a SWCNH concentration of 5 wt.%) and 0.1 mLl IPA during the UV irradiation for 90 min.

To highlight the role of hydroxyl radicals in AMOX photodegradation, Figure 10 shows the UV-VIS spectra of the 98.5 μM AMOX solution containing 0.2 mg/mL of the TiO_2_/SWCNH composite (with a SWCNH concentration of 5 wt.%) and 0.1 mL IPA when the sample is UV-irradiated for 90 min.

**Figure 11:**
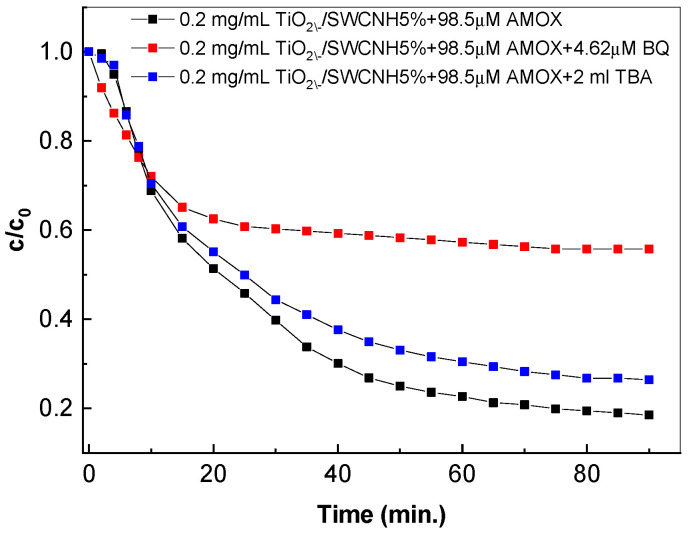
Photocatalytic degradation of the 98.5 μM AMOX aqueous solution in the presence of 0.2 mg/mL TiO_2_/SWCNHs (with a SWCNH concentration being equal to 5 wt.%) before (black curve) and after adding 4.62 μM BQ (red curve) and 2 mL TBA (blue curve).

According to Figure 10, after the UV exposure of the AMOX solution containing the TiO_2_/SWCNH composite and IPA, the degradation efficiency becomes equal to 83.7%, a value that is smaller than 92.4%. This result highlights the role of IPA as a quencher. In order to explore the active species in the photocatalytic degradation of AMOX, we use tert-butyl alcohol (TBA) as a scavenger of •OH and benzoquinone (BQ) for O_2_^•−^. Figure 11 shows the photocatalytic conversion ratios (PCRs), i.e., c/c_0_, of the degradation of the aqueous solution of 98.5 μM AMOX in the absence of scavengers and in the presence of TBA and BQ. 

According to Figure 11, the values of PCRs in the presence of TBA and BQ are smaller than in their absence. A careful analysis indicates that the effect of BQ is more obvious than that of TBA. AMOX degradation was largely induced by the participation of O_2_^•−^ radicals and, to a lesser extent, by the contribution of •OH radicals.

Concerning the kinetics of the photodegradation of the AMOX solution containing the TiO_2_/SWCNH composite, the constant of the degradation rate can be assessed with the following equation:

ln(*A*_0_/*A_t_*) = k × t,

where (i) *A_t_* and *A*_0_ denote the absorbance of the AMOX band localized at 258–278 nm when the time (t) of UV irradiation was between 2 and 90 min, and 0 min, respectively; and (ii) k is the rate constant of the AMOX photodegradation reaction at time (t). 

**Figure 12:**
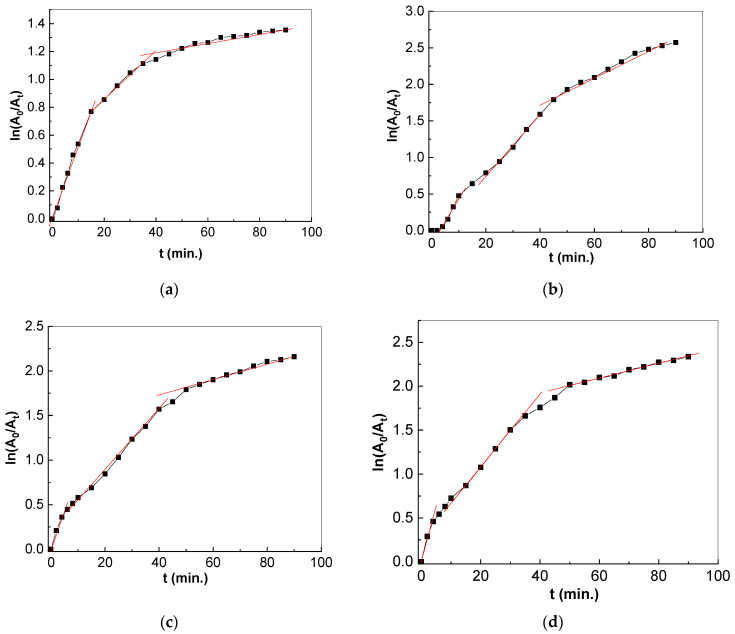
Reaction kinetics of 98.5 μM AMOX aqueous solutions containing 0.2 mg/mL TiO_2_ (**a**) and 0.2 mg/mL TiO_2_/SWCNH composite with a SWCNH concentrations equal to 1 wt.%, (**b**), 5 wt.% (**c**) and 10 wt.% (**d**) under UV light.

According to Figure 12, three linear regions are observed for all catalysts. These correspond to (i) the intermediate products as a result of AMOX photodegradation on the catalyst surface, which are characterized by a rate constant labeled as k_1_; (ii) the photodegradation products as a result of UV irradiation, which has a rate constant labeled as k_2_; and (iii) the surface saturation, which occurs with a rate constant labeled as k_3_.

The rate constant of the AMOX photodegradation reaction in the case of TiO_2_ and the TiO_2_/SWCNH composites with a SWCNH concentration equal to 1 wt.%, 5 wt.% and 10 wt.%, and the linear regression coefficients for the three stages mentioned above, are shown in Table 1. A complex kinetic model of the adsorption process of pharmaceutical compound onto the catalyst’s surface modeled using pseudo-first order rate is reported for the three stages.

Regardless of SWCNH concentration in catalysts mass, the rate constant of the three stages show higher values in the case of the TiO_2_/SWCNH composites in contrast with TiO_2_. This fact indicates a higher loading of SWCNHs onto the TiO_2_ surface. 

## 3. Materials and Methods

TiO_2_, SWCNHs, clavulanate potassium (CLAK, C_8_H_3_KNO_5_), AMOX (C_16_H_19_N_3_O_4_S), p-benzoquinone (BQ) and tert-butyl alcohol (TBA) were procured from Sigma-Aldrich. According to specification sheets, (i) the TiO_2_ sample corresponds to a nano-powder with a particle size < 25 nm and 99.7% trace metals basis and the crystalline phase corresponding to anatase, and (ii) the average length and diameter of the SWCNHs is 40–50 nm and 2–5 nm, respectively, and the diameter of their spherical aggregates is about 100 nm. The purity of isopropyl alcohol (IPA), from SC ATOCHIM SRK, was 99.98%, while the purity of CLAK was ≥98.0%. The purity of the anhydrous TBA and BQ was ≥99.5%. The United States Pharmacopoeia (USP) reference standard of AMOX was used for the above experiments.

In these experiments, the drug Amoksiklav used was from a local pharmacy. One tablet of Amoksiklav contains 875 mg AMOX and 125 mg clavulanate potassium (CLAK). The Amoksiklav tablet core contains anhydrous colloidal SiO_2_, magnesium stearate, microcrystalline cellulose crospovidone and croscarmellose sodium, while the film which covered the core consisted of titanium dioxide (E 171), hydroxypropyl cellulose, ethyl cellulose, triethyl citrate, polysorbate 80 and talc.

The TiO_2_/SWCNH composites were prepared by the solid-state interaction of the commercial TiO_2_ particles with SWCNHs by grinding the two chemical compounds for 15 min. After 15 min., the color of the TiO_2_ powder changed from white to grey. Three TiO_2_/SWCNH composites with SWCNH concentration equal to 1 wt.%, 5 wt.% and 10 wt.% were prepared. 

The characterization by Raman spectroscopy of the three TiO_2_/SWCNH composites, TiO_2_ particles and SWCNHs were performed using a MultiRam FT-Raman spectrophotometer from Bruker, in the backscattering geometry, with a resolution of 1 cm^−1^. 

The FTIR spectra of the three TiO_2_/SWCNH composites and TiO_2_ particles were recorded using a Vertex 80 FTIR spectrophotometer from Bruker, with a resolution of 2 cm^−1^.

The samples have also been investigated by X-ray diffraction (XRD) with a Bruker D8 Advance diffractometer equipped with a Cu tube, which provides X-rays with λ_Kα_ = 1.5406 Å. The data acquisition has been made with a step of 0.4 degrees in 2θ, with a multichannel linear detector giving an equivalent of 177 s/step.

The UV-VIS spectra of the solutions containing AMOX, TiO_2_/SWCNH composites or TiO_2_ particles were recorded using Perkin Elmer Lambda 950 UV-VIS-NIR spectrophotometer at a resolution in the UV-VIS range of 0.05 nm. The photocatalytic properties of the colloidal dispersions of the catalyst for the AMOX photodegradation were prepared by adding the TiO_2_/SWCNH composite to the AMOX aqueous solution, which was ultrasonicated for 5 min. followed by magnetic stirring for 20 min at 25 °C in the dark, until reaching the adsorption–desorption equilibrium of AMOX on the surfaces of the TiO_2_/SWCNH catalysts. To assess the photodegradation efficiency of the catalysts, we will prepare the following: (a) AMOX aqueous solutions with a concentration of 98.5 μM and 197 μM, which are prepared with powder from Sigma-Aldrich; (b) colloidal dispersions of the TiO_2_/SWCNH composites, with a SWCNH concentration of 1 wt.%, 5 wt.% and 10 wt.%; and (c) colloidal dispersions with a concentration of TiO_2_/SWCNH composites in an AMOX aqueous solution equal to 0.1 mg/mL, 0.2 mg/mL and 0.3 mg/mL. For the analysis using Amoksiklav, each tablet was ground for 5 min, dispersed in 100 mL of distilled water under ultra-sonication for 10 min, and filtered to separate the excipients of active compounds, i.e., AMOX and CLAK. To assess the potential of the TiO_2_/SWCNH composites as catalyst for CLAK, a 25 μM CLAK aqueous solution was prepared. For assessing the efficiency of the TiO_2_/SWCNH composites when they are reused in successive photodegradation cycles of AMOX, the protocol published in Ref. [23] was used. The photodegradation process of AMOX was carried out using a lamp with an emission maximum at 367 nm and power of 100 mW. 

## 4. Conclusions

Novel results concerning the photocatalytic properties of TiO_2_/SWCNH composites, prepared by the solid-state interaction of the two constituents, i.e., SWCNHs and TiO_2_, on AMOX degradation are reported. The optical characterization of the TiO_2_/SWCNH composites, highlighted by Raman scattering and FTIR spectroscopy, has demonstrated the following: (i) a variation of the ratio between the intensities of the Raman lines located at 145 and 1595 cm^−1^, which were attributed to the vibrational modes E_g_(1) of TiO_2_ and the graphitic structure of SWCNHs, and a growth of the D band intensity occurring as a result of the defects’ weight increase on the surface of the SWCNHs; and (ii) a gradual increase in the absorbance of the IR bands at 1735 cm^−1^ and 3728 cm^−1^ as a consequence of the C=O and -OH bonds coming from carboxylic groups being generated on the surface of the SWCNHs. The best photocatalytic properties were obtained for the TiO_2_/SWCNH composites, with a SWCNH concentration of 5 wt.%, when 92.4% AMOX removal after 90 min of UV irradiation was achieved. The reuse of the TiO_2_/SWCNH composite, with a SWCNH concentration of 10 wt.%, after six cycles of AMOX photodegradation induces a decrease in the degradation efficiency of 98.5 μM AMOX aqueous solution from 90.3% down to 78%.

## Figures and Tables

**Table 1:** Reaction constants of 98.5 μM AMOX aqueous solutions containing catalysts of either TiO_2_ or the TiO_2_/SWCNH composites with a SWCNH concentration equal to 1 wt.%, 5 wt.% and 10 wt.%.

Sample Name	$k_{1}$ (min^−1^)	$R_{1}^{2}$	$k_{2}$ (min^−1^)	$R_{2}^{2}$	$k_{3}$ (min^−1^)	$R_{3}^{2}$
TiO_2_	0.0053	0.9934	0.018	0.9957	0.0029	0.9594
TiO_2_/SWCNH 1%	0.090	0.9908	0.034	0.9953	0.01	0.9972
TiO_2_/SWCNH 5%	0.072	0.9905	0.0041	0.9941	0.019	0.9943
TiO_2_/SWCNH 10%	0.114	0.9786	0.038	0.9983	0.008	0.9911

## Data Availability

The dataset and analyzed are not publicly available but may be obtained from the corresponding author upon reasonable request.
